# A rare case of cardiac metastatic uterine intravenous leiomyomatosis: A case report

**DOI:** 10.1097/MD.0000000000047553

**Published:** 2026-02-20

**Authors:** Ganghua Yang, Tao Wang, Zehua Tu, Xiaodong Cai, Ruiyu Li, Mengjun Wu, Linling Zeng

**Affiliations:** a Department of Anesthesiology, Xiaolan Clinical Institute of Shantou University Medical College, Xiaolan People’s Hospital of Zhongshan (The Fifth People’s Hospital of Zhongshan), Guangdong Province, P.R. China; b Department of Anesthesiology, Guangdong Provincial People’s Hospital (Guangdong Academy of Medical Sciences), Southern Medical University, Guangdong Province, P.R. China.

**Keywords:** heart dysfunction, Intravenous leiomyomatosis, right atrial mass, tricuspid regurgitation

## Abstract

Intravenous leiomyomatosis (IVL) originating from the uterus with extension into the cardiac chambers is an extremely rare clinical entity, and cases involving severe right-sided cardiac involvement are particularly uncommon; this report aims to document this rare presentation and its management for clinical reference. A patient presented with clinical manifestations of right heart dysfunction, and auxiliary examinations identified severe cardiac involvement secondary to uterine IVL. The patient was diagnosed with uterine intravenous leiomyomatosis with extensive extension into the right cardiac chambers, complicated by very severe tricuspid regurgitation. A combined multidisciplinary surgical approach was performed, including resection of the right atrial mass, tricuspid annuloplasty ring implantation, exploration and resection of the inferior vena cava lesion, total hysterectomy, and bilateral salpingo-oophorectomy. The surgical intervention was successful, and the patient achieved a favorable clinical recovery with good postoperative condition. This is the first reported case detailing this rare and severe presentation of uterine IVL with extensive right cardiac extension. Recognition of this unusual manifestation of IVL is critical for timely diagnosis and the formulation of a comprehensive surgical treatment strategy. Multidisciplinary collaboration is essential for the successful management of such complex cases.

## 1. Introduction

Intravenous leiomyomatosis (IVL) is a rare benign smooth muscle tumor, typically originating from uterine leiomyomas or the myometrium.^[1–3]^ Cases involving intracardiac invasion are exceptionally rare. Preoperative diagnosis remains challenging, and no consensus currently exists regarding its optimal clinical management.^[4,5]^ Surgery constitutes the primary treatment.

Herein, we report a rare case of IVL originating from the uterus with extensive extension into the right cardiac chambers. The patient presented with right heart dysfunction and echocardiography revealed very severe tricuspid regurgitation (TR). Successful management involved a combined surgical approach including resection of the right atrial mass, tricuspid annuloplasty ring implantation, exploration and resection involving the inferior vena cava (IVC), total hysterectomy, and bilateral salpingo-oophorectomy. To our knowledge, this represents the first reported case detailing such a rare and severe combination of findings.

The report was approved by the Institutional Review Board of Guangdong Provincial People’s Hospital (Guangdong Academy of Medical Sciences).

## 2. Case report

A 47-year-old woman was admitted to the hospital with bilateral leg edema and abdominal distension for a year. The patient’s medical history recorded no heart palpitations, breathing difficulties or other symptoms. She had a history of 1 cesarean delivery. Transthoracic echocardiography revealed a large (55 mm × 23 mm), mobile, heterogeneous mass within the right atrium, associated with right ventricular dysfunction and severe TR. Additional findings included mild pulmonary hypertension, moderate mitral regurgitation, and a small-to-moderate pericardial effusion. All relevant biochemical indicators, including hematologic and tumor markers, were normal.

**Figure 1.**
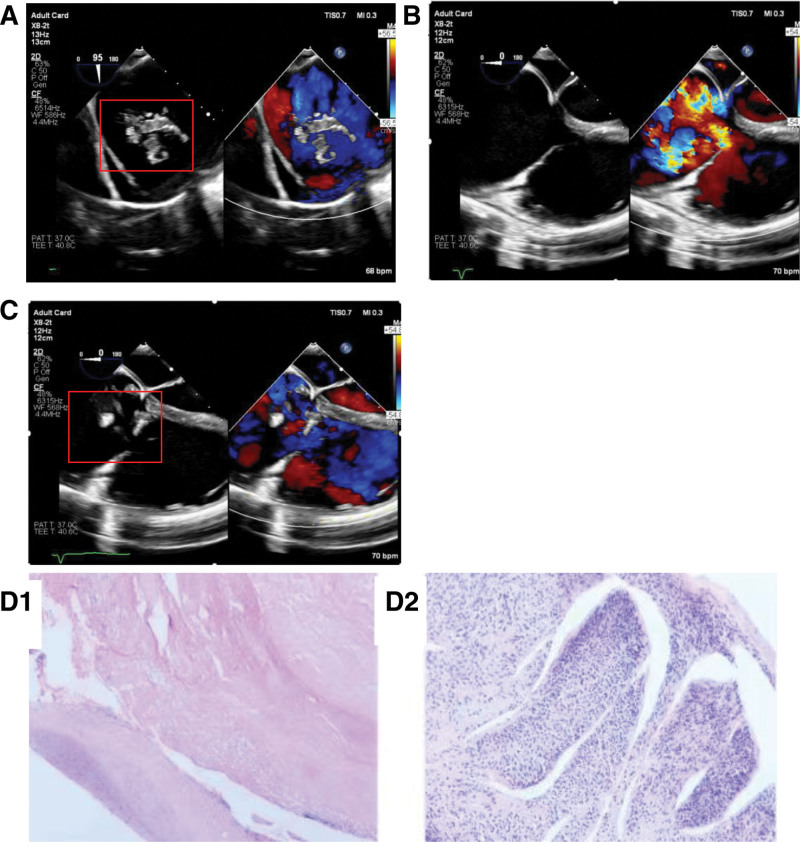
(A) Cardiac mass (red frame) in the right atrium; (B) during systole, severe tricuspid regurgitation; (C) during diastole, a portion of the mass traversed the tricuspid valve, prolapsing into the right ventricle. (D) Under the microscope, the cells in the specimen are spindle-shaped.

Intracardiac tumor resection was performed via median sternotomy utilizing cardiopulmonary bypass (CPB) and mild hypothermia. Preoperative transesophageal echocardiography demonstrated that during diastole, a portion of the mass traversed the tricuspid valve, prolapsing into the right ventricle (Fig. 1A–C). Histopathological examination confirmed the diagnosis (Fig. 1D1 and D2). The histopathology report showed that the Ki-67 positive rate in the immunohistochemical analysis was 1%. The smooth muscle actin (SMA+++), Desmin (+++) and Caldesmon (+++) were positive, while the Melan A, S100, CK, and STAT6 were negative.

**Figure 2.**
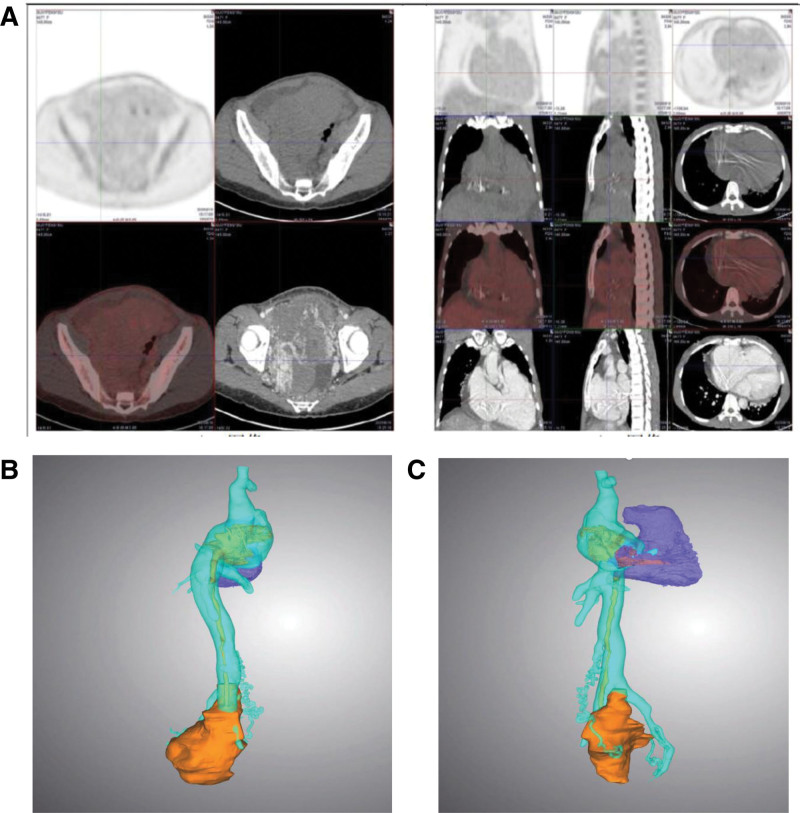
Multiple uterine fibroids: A mass with calcification extending from the right external iliac vein → IVC → right atrium, suggestive of IVL (likely uterine origin). (A–C) No evidence of malignant hypermetabolic lesions elsewhere on whole-body ^18^F-FDG PET/CT. ^18^F-FDG = ^18^F-fluorodeoxyglucose, IVC = inferior vena cava, IVL = intravenous leiomyomatosis, PET/CT = positron emission tomography/computed tomography.

Given the suspected diagnosis of IVL, whole-body positron emission tomography/computed tomography (PET/CT) was performed. This revealed multiple uterine fibroids and masses. Notably, luminal masses within the uterus demonstrated continuous extension into the right external iliac vein, progressing through the IVC, and extending into the right atrium (Fig. 2A–C).

**Figure 3.**
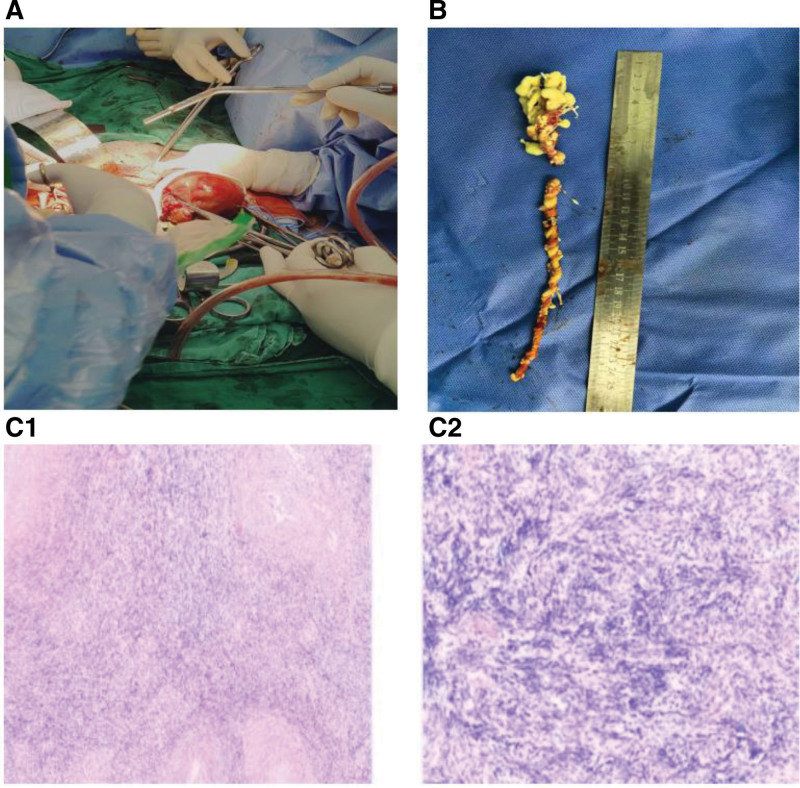
Leiomyoma specimens taken from the right atrium and inferior vena cava (A and B). Under the microscope, histopathological results confirmed intravenous leiomyomatosis (C1 and C2).

The patient underwent a combined surgical procedure involving IVC, along with total hysterectomy and bilateral salpingo-oophorectomy (Fig. 3A and B). Histopathological examination confirmed the diagnosis of intravenous leiomyoma. The tumor was characterized by marked cellularity, morphologically homogeneous tumor cells, and rare mitotic figures (Fig. 3C1 and C2). Ki-67 positive rate immunohistochemical analysis was 1%. The SMA, Desmin, H-caldesmon, Vimentin, and CD34 were positive, while the Melan A, S100, CK, and STAT6 were negative.

## 3. Discussion

While most IVL patients have a history of hysterectomy or preceding uterine fibroid symptoms,^[6,7]^ our patient presented with bilateral leg edema and abdominal distension without prior surgery. Notably, a literature review indicates 64% of reported cases involve women with a hysterectomy history ranging from 6 months to 20 years prior to IVL diagnosis.^[6]^ Therefore, in patients scheduled for uterine leiomyoma surgery, symptoms like chest tightness, dyspnea, or lower limb edema should raise suspicion of IVL with IVC and cardiac extension. Preoperative echocardiography is indispensable in such cases, as it effectively delineates tumor relationships with cardiac chambers, valves, and other structures.

A multidisciplinary team comprising gynecology, cardiothoracic surgery, anesthesiology, and intensive care specialists evaluated therapeutic strategies. Due to right heart dysfunction (right atrial diameter: 82 mm) and severe TR, single-stage resection was initially deemed suboptimal for this patient. Notably, although severe TR typically correlates with comorbidities including heart failure, pulmonary hypertension, and arrhythmias, this patient was remarkably asymptomatic preoperatively – reporting no palpitations, dyspnea, or functional limitations. Transthoracic echocardiography revealed diastolic prolapse of a tumor fragment through the tricuspid valve into the right ventricle, we thought a mechanical phenomenon likely exacerbated TR severity. Given the patient’s relatively young age (47 years) and preserved multisystem organ function, we established rigorous perioperative protocols through multidisciplinary collaboration to optimize surgical conditions.

Severe TR represents a progressive condition linked to significant morbidity, impaired quality of life, and elevated mortality.^[8–10]^ This patient exhibited a rare constellation of severe pathologies: right ventricular dysfunction, severe TR, mild pulmonary hypertension, and moderate mitral regurgitation. Consequently, perioperative management necessitates comprehensive understanding of right heart pathophysiology and individualized anesthetic planning.

Key hemodynamic objectives include: Optimizing preload to sustain adequate cardiac output while avoiding volume overload exacerbation (target central venous pressure: 8–10 mm Hg).^[8,9]^ Utilizing intraoperative transesophageal echocardiography (TEE) for real-time assessment of valve integrity, right ventricular performance, and dynamic hemodynamic changes.^[9]^ Selecting vasopressin as the preferred vasopressor due to its minimal impact on pulmonary vascular resistance and direct coronary perfusion benefits.

To our knowledge, this represents the first reported case of a female patient exhibiting this rare constellation of severe manifestations – significant right heart dysfunction, severe TR, intracardiac tumor extension, and uterine involvement. Prior publications, such as a symptomatic 50-year-old female with IVL extending from the right ovarian vein through the common iliac vein to the IVC and right atrium, lacked concurrent right ventricular impairment or severe valvulopathy. This case expands the clinical spectrum of IVL and highlights critical implications for managing intracardiac involvement.

## Author contributions

**Data curation:** Ganghua Yang.

**Formal analysis:** Linling Zeng.

**Investigation:** Tao Wang.

**Methodology:** Xiaodong Cai.

**Resources:** Zehua Tu.

**Supervision:** Ruiyu Li, Mengjun Wu.
